# Nurses Experiences Caring for Critically Ill Children Receiving Non-Invasive Ventilation in Intensive Care Units – A Qualitative Study

**DOI:** 10.1177/23779608261476493

**Published:** 2026-09-22

**Authors:** Tone Magnussen, Outi Koistinen, Anna Apfelstedt, Monica Evelyn Kvande, Brith Andresen

**Affiliations:** 1 155319 Department of Master and Postgraduate Studies, Lovisenberg Diaconal University College, Oslo, Norway; 2 Department of Postoperative and Critical Care Nursing, 155272 Oslo University Hospital Rikshospitalet, Oslo, Norway; 3 Department of Cardiothoracic Surgery, 155272 Rikshospitalet, Oslo University Hospital, Oslo, Norway; 4 Department of Anaesthesiology and Surgery, 60519 University Hospital of North Norway, Tromsø, Norway

**Keywords:** acute respiratory distress, critically ill children, paediatric intensive care unit, non-invasive ventilation, nurses experiences

## Abstract

**Introduction:**

Acute respiratory distress is a common cause for admission of children to intensive care units, often caused by infections. Non-invasive ventilation is used in the treatment of critically ill children with respiratory failure and is a preferred treatment strategy. Objective: We aimed to explore nurses’ experiences of non-invasive ventilation in critically ill children in intensive care units.

**Methods:**

A qualitative study with an exploratory design was conducted. Data were collected through 13 semi-structured interviews in five intensive care units in five hospitals in Norway. Data were analysed using reflexive thematic analysis and reported according to the Consolidated Criteria for Reporting Qualitative Research checklist. This study was approved by the Norwegian Agency for Shared Services in Education and Research (SIKT) in 2023 (approval no. 826313) prior to data collection and by the hospital’s Data protection officers of five different hospitals. All participants provided informed consent.

**Results:**

Three themes emerged: 1) Balance between interaction and treatment; 2) Teamwork makes a difference; and 3) From disturbance to resource – collaboration with parents. Nurses experienced that these aspects helped them to create safety and trust with children and parents, which benefited non-invasive ventilation.

**Conclusion:**

Early trust, clear communication and coherent collaboration were highlighted as essential for the success of non-invasive ventilation. Lack of time and uncertainty about equipment for non-invasive treatment could have a negative impact. The nurses’ years of experience made a difference. This study underscored a need for improvement in both communicative and technical skills and strengthen a coherent interprofessional collaboration.

## Introduction

Acute respiratory distress, often resulting from infections is a common cause for children being admitted to intensive care units (ICUs) (Ames et al., 2024). The paediatric airways are smaller in diameter, shorter and less developed than in adults. Infections can seriously compromise a child’s airways with even mild swelling. Children are highly susceptible to respiratory infections as their respiratory system is less adaptable to cope with the changes in ventilation. Infections that affect a child’s airway can impair oxygen exchange, reduce alveolar ventilation and ultimately exhaust the child due to an increased workload on the respiratory muscles (Kuzucu et al., 2025).

## Review of the Literature

Non-invasive ventilation (NIV) is a promising treatment strategy used in intensive care units to treat children with respiratory failure (Feng et al., 2025). In this treatment, oxygen is delivered via a mask with a positive airway pressure (Boghi et al., 2023). Compared to mechanical ventilation, NIV is associated with fewer complications and a lower need for sedative medication. Clinical evidence supports widespread use of NIV for multiple indications (Emeriaud et al., 2023). In a recent meta-analysis investigating the efficacy of NIV in preventing intubation in pediatric acute respiratory failure, in fifteen studies compromising more than 10,000 pediatric patients (Sajid et al., 2026), NIV was associated with a significant reduction in the need for intubation, with pooled estimates indicating a 21% relative reduction in intubation risk (risk ratio [RR] = 0.79, 95% confidence interval [CI] 0.63–1.00). Its success was strongly dependent on early initiation, careful patient selection, and close monitoring within experienced PICU settings.

The benefit of NIV in severely hypoxemic patients remains unclear, as does its efficacy in conditions such as bronchospasm and asthma (Baker et al., 2023). Patients with severe hypoxemia or ARDS are identified as major-risk groups in whitch the use of NIV are rarely recommended (Emeriaud et al., 2023). Various procedures and devices are available for paediatric NIV, with varying success rates depending on the age and clinical condition of the child (Cabrera & Tapia, 2025).

However, there are also potential risks associated with NIV treatment (Emeriaud et al., 2023). These risks include delayed endotracheal intubation when required, as well as air leaks around the mask, which can lead to inadequate positive end-expiratory pressure. Obtaining a correctly fitting mask for the child can be a major challenge and is equally important for implementation (Cassibba et al., 2023). Restlessness, discomfort due to the mask and lack of cooperation are described as possible causes of failed NIV treatment (Bedi et al., 2023). In addition, NIV can be difficult for children to tolerate unless they are suffering from severe dyspnea (Cassibba et al., 2023). Unsuccessful delivery of treatment may lead to a deterioration in the child’s condition. This can potentially lead to the need for endotracheal intubation, which should be avoided if possible (Boghi et al., 2023).

Children treated with NIV require continuous observation and the expertise of healthcare professionals (Carroll et al., 2023). Nurses have a major responsibility in initiating NIV and in the ongoing assessment of the child’s clinical status during treatment (Cassibba et al., 2023). Paediatric patients have challenges and barriers related to treatment in terms of communication and understanding. Creating a safe and predictable environment for critically ill children in the ICU is essential. This requires healthcare professionals to act on and implement interventions based on nursing expertise, situational awareness and experience in paediatric care (Chong et al., 2025). Family-centred care encompasses the care of the family as well as the patient and is essential for both the child and the family. Parents play an important role in comforting the child so that he or she feels safe and cooperates with treatment (Alqarawi & Alhalal, 2024). Children admitted to hospital can exhibit high levels of stress, anxiety and help-seeking behaviour and need a lot of support from parents and healthcare professionals to feel safe (Claridge & Powell, 2023).

The knowledge and guiding data on NIV are primarily based on healthcare professionals’ experiences of treating adult patients with NIV (Baker et al., 2023). Despite some similarities between adults and paediatric patients, the extrapolation of adult data to paediatrics should be critically questioned due to various factors such as differences in physiology and anatomy, cognitive maturity, and level of verbal communication (Barratt et al., 2023; Panetti et al., 2024). Currently, there is a research gap and a need for greater insight and understanding of ICU nurses’ experiences caring for critically ill children receiving NIV. More evidence-based studies are needed to fill this gap.

Objectives of this study was to explore nurses’ experiences with non-invasive ventilation in critically ill children in the intensive care unit.

## Methods

### Study Design

Researchers used an exploratory design in this qualitative study. This design is suitable for investigating and developing new knowledge on topics that are limited in clinical nursing research. The Consolidated Criteria for Reporting Qualitative Research (COREQ) checklist was used as a guide for this study (Tong et al., 2007).

### Participants and Setting

Participants were recruited from five intensive care units in five hospitals in Norway treating children with NIV. We used a purposive sampling strategy and included nurses who had at least one year of experience with NIV treatment of children aged 1-12 years.

Ward managers recruited nurses by distributing study information about the study in the participating units. Interested nurses contacted their unit manager, who referred them to the first authors (TM, OK, AA). Thirteen nurses agreed to participate in the study (Table 1).

**Table 1.** Clinical Characteristics of Participants

Clinical characteristics of participants							
Gender	N = 13	Age	N	Work experience (years)	N	Ward units	N
Female/Male	13/0	25-35	1	1-5	1	Pediatric units	9
​	​	35-45	6	5-10	1	Mixed units	3
​	​	45-55	2	20-30	2	​	​
​	​	55-65	4	30 or more	2	​	​

The study was approved by the Norwegian Agency for Shared Services in Education and Research (SIKT) in 2023 (approval no. 826313) and by the hospital’s Data protection officers of five different hospitals.

### Data Collection

Data were collected through individual interviews between November and December 2023. 13 interviews were conducted, lasting 25-50 minutes. Nine of the interviews were conducted online and four face-to-face in a quiet room at their workplace. Data were collected both through personal interviews and via Zoom to include perspectives from multiple intensive care units in Norway to enhance the credibility and transferability of the findings. The interview guide consisted of 12 open-ended questions with the option of follow-up questions. This gave participants the opportunity to elaborate on perspectives and topics beyond the prepared interview questions and thus raised issues that the interviewers had not addressed (Polit & Beck, 2021). A pilot interview was conducted to ensure that the questions in the interview guide were relevant and understandable. Afterwards, two questions were modified to allow for a broader approach. When necessary, leading questions were used to achieve a higher variation and quality of the collected data (Polit & Beck, 2021). Each interview was conducted by two of the three first authors (TM, OK, AA). One author interviewed the participants while the other took notes. The third author transcribed the audio recordings. The roles of interviewer, note-taker and transcriber were alternated throughout the data collection. This was done to constantly check the possible biases of the researchers and prevent them from interfering with the interview process. The intention was to maintain an open mind and an enquiring mindset.

There were no personal or professional connections between the interviewers and the participants, apart from two participants who worked at the workplace of one of the first authors. The two interviews were conducted by the two first authors who had no relationship with the respective participants. Sample size was determined by the study’s aim and by the empirical material required to illuminate the phenomenon in a meaningful way. A relevant, experience-near sample gave and rich and varied data sufficient to provide insight into how the phenomenon appeared and was understood by the participants (Malterud, 2025; Polit & Beck, 2021). The interviews were recorded.

### Analysis

The interviews were transcribed verbatim and analysed using a reflexive thematic analysis that followed the six phases described by Braun and Clarke: familiarizing; coding; generating initial themes; reviewing and developing themes; refining, defining and naming themes; and writing the report (Braun & Clarke, 2021). The first authors (TM, OK, AA) read the interviews individually several times to familiarize themselves with the data. During this phase, the authors noted interesting and relevant findings and patterns. Participants’ statements and comments were coded, and the codes were then grouped into categories. These categories formed the focus of the analysis, and the emerging categories became the basis for defining several preliminary themes. This process followed an iterative method of analysis, which was chosen to ensure the validity of the defined patterns while discovering new ones (Braun & Clarke, 2021). Themes that did not fit were reconsidered, edited or discarded. The final themes were then condensed into a text and formed the findings of this study. Relevant quotations from the transcripts were selected to illustrate the text elements. Finally, the transcripts were reread to ensure that the interpretations were valid representations of the original transcripts (Table 2).

**Table 2.** Examples of Extractions from the Qualitative Analysis

Interview guide	Follow-up questions	Narrative	Code	Subtheme	Theme
Examples of extractions from the analysis					
What role do the parents play in the preparations?	Is the child affected by how the parents are doing?	*They [parents] are often stressed for a reason, and I often find the most stressed parents have been through a dramatic admission. That they have been very scared due to their child being ill, maybe for the first time. In those cases, there might be a lot of comforting of the child, that is not comforting at all, and the parents may include themselves in the treatment in a not so appropriate way (Participant 7)*	Frightened or stressed parents can pose barriers to efficient treatment	Parents approach to their child	From disturbance to resource – collaboration with parents
	How do you think parents influence the child?	*If the parents are also scared, not knowing what’s going on, then they might signal fear to the child, and it leads to them not being the safe support they are supposed to be. But of course, many parents haven’t been through having their child put on NIV, so it’s a completely new thing for them too. Therefore, it’s really important that we spend some time informing them about the situation and what’s going to happen. That we stay calm, don’t run back and forth, and that we demonstrate control (Participant 12)*	Uncertainty within parents can cause fear in children		
	How do you use parents during treatment?	*It can often be that the parents, for example, help to put on the mask (…), if they feel comfortable doing it. (…) I believe being able to include the parents in the treatment, is good for the child. Because it’s the parents who are the comfort and the safety for the child” (Participant 1)*	NIV entails parental training and guidance responsibilities Parents as a resource	Interaction between nurses and parents	
Example of findings extracted from the analysis					
What role do the parents play in the preparations?	Is the child affected by how the parents are doing?	*They [parents] are often stressed for a reason, and I often find the most stressed parents have been through a dramatic admission. That they have been very scared due to their child being ill, maybe for the first time. In those cases, there might be a lot of comforting of the child, that is not comforting at all, and the parents may include themselves in the treatment in a not so appropriate way (Participant 7)*	Frightened or stressed parents can pose barriers to efficient treatment	Parents approach to their child	From disturbance to resource – collaboration with parents

## Results

Three themes were identified: 1) Balance between interaction and treatment; 2) Teamwork makes a difference; and 3) From disturbance to resource – collaboration with parents. Findings in the first two themes focused mainly on the interaction between the child and the nurse, even when the parents were present and involved. The third theme focused on the interaction between the nurses and the parents.

### Theme 1 - Balance Between Interaction and Treatment

Nurses in this study revealed that it was a great challenge to build a good relationship with the child and manage NIV at the same time. Nurses expressed a desire to provide high quality of NIV while responding to the individual needs of the child. The invasive nature of NIV can compromise certain needs of the child, such as comfort, closeness to parents, pain relief, and adequate nutrition. Nurses stressed the importance of being able to communicate and interpret the child’s behaviour when initiating and managing NIV.

I often feel like they realise that this [NIV treatment] is easier, at least in the beginning. And as they recover, they start to fight the mask a bit more. But I feel like they often realise, “Wow, this was actually more comfortable” as they put the mask on, even though it’s weird to have this thing on their face. And then they calm down. And then of course some are completely opposite, they really feel like, this makes a lot of noise, I feel strange breathing in it, it has to come off now. (Participant 7)

Establishing a good relationship with the child enabled nurses to tailor the treatment approach based on age and individuality. Nurses organized and grouped procedures to minimize the disturbance of the child and minimized their verbal communication and information between procedures. Nurses expressed that being able to focus under pressure and tolerate seeing a child in distress and have the patience to wait until the child calmed down were essential for succeeding in NIV.

I remember (…) she [the child] came in with acute respiratory infection (…) and she was very sick. (…) The most important thing in that case, was us having patience, because it took some time before the treatment had an effect on her. (…) She had 87- 88 [percentage] in saturation, and I just sat there thinking “God, is it going up or down…”, with almost maximal effort on the oxygen. Then slowly but surely, one number at a time went up and we saw that she became relaxed (…). So, it was one of those success stories. She would have ended up being intubated if it wasn’t for experienced nurses looking after her. (Participant 9)

Nurses expressed a feeling of satisfaction, competence, and professional pride when children tolerated NIV and improved respiratory distress avoiding intubation.

Nothing is better than when a child comes in with severe respiratory distress, (…) considered for intubation, but you come through with NIV-treatment. That is satisfying as a nurse, because there are so many nursing interventions involved in NIV-treatment. (Participant 8)

Nurses felt a strong sense of responsibility to prevent treatment failure and avoid causing potential trauma to the child, which they associated with limited pediatric experience and infrequent exposure to NIV. NIV-treatment that failed to meet expectations often had a negative impact on the nurse.

It was hard for us [nurses], to see that she [the child], was struggling as much as she did. But they didn’t want to intubate either (…). So, it was a challenging case, because we didn’t quite reach our goal, and she was like a fish out of water. (Participant 5)

### Theme 2 - Teamwork Makes a Difference

Nurses in this study emphasized the importance of interprofessional teamwork to prioritize the child’s wellbeing throughout all phases of NIV and to enchance trust and collaboration with parents. The atmosphere during transfer from the ward or admission to the ICU was sometimes described as chaotic and stressful, often when they were unplanned and there was a rapid deterioration in the child’s condition. This forced a focus on rapid implementation of NIV rather than focusing on communication with the child.

They [the children] often come to us [the ICU] in a rush and in a pretty bad condition. So you only have a short window of time to reassure the child, and you also have to reassure the parents. (…) It is of course a challenge to calm the child in a short time, because it often develops over time. (Participant 3)

Working with colleagues in the ICU helped the ward nurse to prepare the equipment and to keep an eye on the child. A calm and controlled demeanour and time to communicate and focus on the child were seen as important for successful initiation of NIV. Involving colleague nurses was also seen as crucial to successfully build a relationship with the child providing a sense of safety. This seemed to correlate with the child’s acceptance of NIV. The opposite would lead to challenges, as one nurse explained:

‘When they come to us [to the ICU], it’s often necessary to get them quickly on NIV, and I often experience that the preparation of the child is given lower priority, which can make the next few days, or at least the next few hours, more difficult. (Participant 7)

They [the ward nurses] often had the opportunity to spend some time with us and they often know the child. (…) They have already built up a relationship and benefit from being familiar with both the child and the parents, so we don’t have to start from scratch to get to know each other. I think it’s great to have them there and I use the ward nurses to the best of their ability to gather information about what they have learned about the child and the parents so far and what we should use in the future. (Participant 4)

Nurses in this study reported that collaboration with the ward nurses during the transfer to the ICU was perceived as positive and safe for the child because ward nurses were a ‘familiar face’ in the ICU. Ward nurses also provided the nurses in the ICU with information regarding the child’s and parents’ preferences. Nurses explained that this made it easier to act in the best interests of the child and to better understand the needs of the child and parents when they were transferred to ICU. Collaboration with doctors was also described as essential. When experienced doctors were present and available to discuss the treatment of the child, nurses felt reassured knowing that the doctors would take medical responsibility and act when necessary. However, nurses were present bedside and closest to the child and stated that decisions were often guided by their clinical experience and observations of changes in the child’s condition.

You depend on having doctors who are also experienced and hear and see the same as you (…). Because we need to be on the same page about things. I, for certain, don’t want to be the only one who is too eager to make this [NIV treatment] work, and then ending up hitting the rock bottom [with the child] when they have to intubate. (Participant 9)

Nurses in this study highlighted that collaboration among healthcare professionals in the ICU positively contributed to ensure continuity of care for the child. When healthcare professionals agreed on and followed the same plan for NIV, it was easier to synchronize when unexpected events occurred.

It matters which nurses and doctors are on duty. There are definitely some doctors and nurses who are a bit more “trigger happy” [eager] to take the mask off, or very quick to put it back on. (…) I feel like this is the most important factor in how long the children end up having NIV. (Participant 7)

### Theme 3 - From Disturbance to Resource – Collaboration With Parents

Nurses emphasized that family-centred care was essential for successful NIV, children both needed and were entitled to have their parents present during NIV. Nurses highlighted the importance of building trust in parents involving and supporting them in comforting their child to create a sense of safety for the entire family. To achieve trust and collaboration, nurses met with individual parents, informed them and guided them in their role as caregivers in the ICU. If parents were scared and uncertain about what is happening, they might unintentionally transfer their anxiety to the child, preventing them from providing the sense of safety. Most parents did not have any experience with their child being connected and treated with NIV. So, it was really important to dedicate time to inform them about the situation and what’s going to happen.

That we stay calm, don’t run back and forth and that we demonstrate control. (Participant 12)

They [parents] are often stressed for a reason, and I often find the most stressed parents have been through a dramatic admission. That they have been very scared due to their child being ill, maybe for the first time. In such cases, the child may be comforted a lot, which is actually not comforting at all, and the parents may participate in the treatment in an inappropriate way. (Participant 7)

Some nurses expressed that they were sometimes unable to calm the parents, no matter how much they informed and supported them. Nurses explained that the parents’ stress was a reaction to their fear that their child might die.

I definitely think it [dramatic admissions] has an impact on how easy it is to achieve good treatment and collaboration with parents. (…) When you have parents who’ve had an experience where they thought “my child is dying”, I find it hard to collaborate with the parents afterwards. They might feel taken good care of and all, but they’ve had such a fright that it makes it hard to make changes in treatment or try out something new. (Participant 7)

Nurses expressed that it was crucial to inform parents about what was considered improvement and signs of recovery and experienced that parents were often more anxious than the child before and during NIV. Guiding parents on how to distract and comfort their child was highlighted as crucial. Delineating roles was a responsibility that nurses needed to discuss and emphasize with parents, especially in situations where parents were stressed.

We try to support the parents so that they can remain calm in the situation, because if they get stressed, the children get stressed too. (…) These children with respiratory distress, they use so much energy just by breathing that crying or anxiety makes the situation even more exhausting, and result in the child to collapse on the spot. So, it’s really important that it is calm around them. (Participant 10)

Some nurses emphasized that supporting parents also involved helping them to avoid exhaustion. This could encourage parents to take breaks from sitting by their child, to eat, take a rest, go for a walk etc. Nurses explained that they adapted themselves and their approach to each individual family, facilitating a safe environment and space in which to perform NIV. Parental involvement through two-way communication and interaction, where parents could share information about the child’s personality, expressions and preferences, was seen as an essential aspect of creating a trusting relationship. Several nurses highlighted this as a key aspect of delivering personalized care and treatment for the entire family.

Parents’ assessments are very important and that they feel heard. That they can share assessments with you, like “What do you think?”. Ask them so that they feel involved, heard and listened to. It is very important to build trust and make them realize the importance of what we do. (Participant 9)

Nurses explained that parents were encouraged to participate in specific tasks such as changing diapers and holding the child during NIV. This was essential for parents to feel included in their child’s care. Parents often needed guidance and reassurance on how to perform these tasks, while the nurse remained responsible for clinical tasks such as suctioning and adjusting the mask. This helped to reinforce the parents’ sense of control over their child and gradually gain confidence in caring for their child when being treated with NIV. However, nurses assessed the parents’ involvement in tasks and considered whether the parents were able and comfortable with being involved in their child’s care.

It can often be that the parents help to put the mask on, for example (…), if they feel comfortable doing so. (…) I believe that it is good for the child if you can involve the parents in the treatment. Because it is the parents who give their child comfort and security. (Participant 1)

## Discussion

The aim of this study was to explore nurses’ experiences with NIV in critically ill children. Three themes were identified. Each theme describes how nurses communicate and interact with children and their parents to build a trusting relationship.

Working with paediatric patients and building a trusting relationship can be challenging due to the uncomfortable procedures which must be performed and children’s common reservations about strangers (Sabetsarvestani & Geçkil, 2024). Finding ways to communicate and bond with the child is something nurses must establish based on competence and experience. Nurses who succeeded in building a trusting relationship with the child, needed to be creative, motivated, open and empathic (Chong et al., 2025).

Several nurses in our study highlighted the importance of taking time to build a relationship with the individual child, however, nurses in the PICU and ICU face time-constraints that can interfere with optimal communication and collaboration with families (Chong et al., 2025). Since children often become anxious in hectic hospital environments, it is important to minimize negative stimuli to calm down the child by taking time to communicate and inform the child.

Nurses with extensive practical and personal experience in paediatric nursing demonstrated a greater ability to manage procedures and quickly build relationships with both the child and their parents (Cho et al., 2025). This clinical experience also appeared to improve nurses’ ability to correctly interpret the child’s verbal and nonverbal communication (Chong et al., 2025). Nurses in our study highlighted that lack of confidence in performing NIV was often due to insufficient practical training and experience in performing procedures. Other studies reported that novice nurses felt insecure due to a lack of experience, making it difficult to anticipate situations when caring for the child and trust their own judgment (Cho et al., 2025; Chong et al., 2025; Masterson et al., 2024).

Theoretical knowledge, practical skills and close supervision are essential for successful NIV implementation and for preventing and detecting treatment related complications (Carroll et al., 2023), and have shown to significantly increase the confidence of inexperienced paediatric nurses (Zhang et al., 2025). Guidelines and available protocols are important to deliver NIV treatment safely and effectively (Sajid et al., 2026). Nurses in our study expressed that a lack of practical training and protocols could lead to treatment reluctance among nurses.

Clear communication and a shared treatment plan within the interprofessional healthcare team can improve the quality of care for the child. Team collaboration is crucial in the treatment of critically ill children due to the complexity of the treatment and the many different tasks that need to be implemented. Well-established and functioning teamwork had a positive impact on the care and treatment of the child (Costello et al., 2021).

Nurses in our study highlighted that collaboration with parents was important and facilitated bonding with the child and positively influenced the success of NIV treatment. This is consistent with research that have found that parent proximity is beneficial as parents contributed to a safe environment (Alqarawi & Alhalal, 2024). In contrast, parents who feel insecure in the situation or question the competence of the caregiver may make it difficult for the caregiver to restablish a good relationship with the child (Masterson et al., 2024).

Nurses in our study experienced parents being stressed when their child needed NIV and highlighted the importance of reassuring and guiding them as supportive partners rather than seeing them as a disruption. Research has shown that providing clear information about the treatment and supporting parents in understanding their child’s situation can have a calming effect (MacKay et al., 2024). Lack of information led to uncertainty and despair. Parents then often trusted their own perception of the situation rather than understanding the complex nature of treatment and treatment changes, consistent with Burgess et al. (2021). Studies found that nurses adjusted their information according to how much they trusted the parents, with the intention of protecting parents from feeling overwhelmed and less stressed (Cheon et al., 2024; Masterson et al., 2024).

Nurses in our study emphasized that two-way communication, individualized information, and clear role delineation between nurses and parents are appropriate tools to make parents a resource. In situations where two-way communication took place, parents felt involved and supported participation in their child’s care. This is supported by research indicating that such tools can enhance collaboration and foster trust in line with MacKay et al. (2024) describing the importance of providing nurses with direction and strategies on how to develop and maintain trust in children and caregivers to provide safe and high-quality care. However, nurses in our study experienced that it was challenging to find a balance and not overwhelm the parents while guiding and encouraging them to take breaks for their own well-being.

Supporting parents to contribute to the child’s treatment by gaining insight and information about the child was beneficial to the relationship. Tavallali et al. (2025) highlighted nurses recognizing the need for individuality in care of each family and the importance of building a balanced partnership to achieve high quality care. When the relationship between the nurses and the parents was well established, the child was more likely to trust the nurse, which eased the separation from the parents. This allowed the parent to rest and attend to basic needs, enchaning them to be a better resource for their child (Poole et al., 2024). This suggests that building a trust-based relationship with both the parent and the child is crucial to the successful implementation of NIV.

### Strengths and Limitations

The recruiting process might have influenced the research process since the unit managers promoted participants who represented the unit positively. To avoid bias, the researchers placed information letters about the study in the units, giving nurses from the different units the opportunity to volunteer rather than being promoted by the managers. Nurses who volunteered engaged with the topic, which contributed to rich and diverse data. The participants in this study were from all four regional health authorities in Norway and may be a strength. However, it might also be a bias since the participants might consist of nurses who are committed and frequent contributors to studies and therefore not representative of the whole population. Although there were no male participants, the diversity among the participants may be a bias and a limitation that represents the reality of practice and promotes transferability in the study. The included participants had a variety of work experience. For example, only one of the participants worked on a general paediatric unit, while the others worked on paediatric or mixed ICUs.

### Implications for Clinical Practice

This study highlights the need for strong interprofessional collaboration and adequate support for nurses to improve outcomes in NIV-treatment for critically ill children. Providing sufficient time and resources is essential to help nurses establish trusting relationships with children and their families, which is a key to treatment success. A calm and supportive care environment should be prioritized to foster trust and treatment acceptance. Additionally, addressing the emotional burden nurses experience when treatment fails is crucial for maintaining their well-being and sustaining high-quality care.

## Conclusion

Nurses experienced the establishment of considerate interactions, cohesive collaboration and trusting relationships as essential aspects of successful NIV-treatment in critically ill children.

Collaboration within the interprofessional healthcare team was highlighted as crucial and contributed to better time management and outcomes in NIV-treatment. However, nurses reported that they bore a great responsibility when NIV treatment failed. Given the lack of time, nurses felt insecure and unable to focus on a reassuring and trusting relationship with the child and parents. Successful treatment, seen as well-informed parents, and a child who accepted treatment and recovered, had a positive impact on the nurses, enhancing feelings of satisfaction, competence and professional pride. Maintaining a calm and controlled atmosphere benefited the child and their parents in building trust.

## Data Availability

Data collected in this study are not available without special permission from the Data Protection Officer of the participating hospitals.*

## References

[bibr1-23779608261476493] Alqarawi N. Alhalal E. (2024). Factors affecting family-centered care practice by nurses: A systematic review. Journal of Pediatric Nursing, 78, 158–171. 10.1016/j.pedn.2024.06.008 38944912

[bibr2-23779608261476493] Ames S. G. Maddux A. B. Burgunder L. Meeks H. Fink E. L. Meert K. L. Zinter M. S. Mourani P. M. Carcillo J. A. Carpenter T. Pollack M. M. Mareboina M. Notterman D. A. Sapru A. Eunice Kennedy Shriver National Institute of Child Health and Human Development Collaborative Pediatric Critical Care Research Network CPCCRN . (2024). Healthcare Burden and Resource Utilization After Pediatric Acute Respiratory Distress Syndrome: A Secondary Analysis of the Collaborative Pediatric Critical Care Research Network Acute Respiratory Distress Syndrome Study. Pediatric Critical Care Medicine, 25(6), 518–527. 10.1097/pcc.0000000000003476 PMC11178270 38445974

[bibr3-23779608261476493] Baker A. Beardsley A. L. Leland B. D. Moser E. A. Rowan C. M. Cristea A. I. (2023). Predictors of Failure of Non invasive Ventilation in Critically Ill Children. Journal of Pediatric Intensive Care, 12(3), 196–202. 10.1055/s-0041-1731433 PMC10411242 37565011

[bibr4-23779608261476493] Barratt M. Bail K. Paterson C. (2023). Children living with long-term conditions and their experiences of partnership in nursing care: An integrative systematic review. Journal of Clinical Nursing, 32(13-14), 3185–3204. 10.1111/jocn.16429 35768938

[bibr5-23779608261476493] Bedi P. K. DeHaan K. Ofosu D. Olmstead D. MacLean J. E. Castro-Codesal M. (2023). Predictors of NIV-related adverse events in children using long-term noninvasive ventilation. Pediatric Pulmonology, 58(12), 3549–3559. 10.1002/ppul.26689 37701936

[bibr6-23779608261476493] Boghi D. Kim K. W. Kim J. H. Lee S.-I. Kim J. Y. Kim K.-T. Ambrosoli A. Guarneri G. Landoni G. Cabrini L. (2023). Noninvasive Ventilation for Acute Respiratory Failure in Pediatric Patients: A Systematic Review and Meta-Analysis. Pediatric Critical Care Medicine, 24(2), 123–132. 10.1097/pcc.0000000000003109 36521191

[bibr7-23779608261476493] Braun V. Clarke V. (2021). Thematic analysis: A practical guide. Sage Publications.

[bibr8-23779608261476493] Burgess A. Wilcoxon L. Rushworth I. Meiser-Stedman R. (2021). Meta-analysis found high rates of post-traumatic stress disorder and associated risk factors in parents following paediatric medical events. Acta Paediatrica, 110(12), 3227–3236. 10.1111/apa.16113 34534369

[bibr9-23779608261476493] Cabrera A. J. Tapia I. E. (2025). Non-invasive ventilation in pediatrics: a narrative review. Part 1: conventional non-invasive ventilation. Paediatric Respiratory Reviews. 10.1016/j.prrv.2025.11.004 41314948

[bibr10-23779608261476493] Carroll C. L. Napolitano N. Pons-Òdena M. Iyer N. P. Korang S. K. Essouri S. Second Pediatric Acute Lung Injury Consensus Conference of the Pediatric Acute Lung Injury Sepsis Investigators Network . (2023). Noninvasive Respiratory Support for Pediatric Acute Respiratory Distress Syndrome: From the Second Pediatric Acute Lung Injury Consensus Conference. Pediatric Critical Care Medicine, 24(Supplement 1 2S), S135–S147. 10.1097/pcc.0000000000003165 36661442

[bibr11-23779608261476493] Cassibba J. Fumagalli A. Alexandre A. Chauveau A. Milési C. Mortamet G. (2023). How crucial is the role of pediatric critical care nurses in the management of patients with noninvasive ventilatory support. Pediatric Pulmonology, 58(9), 2678–2680. 10.1002/ppul.26549 37283239

[bibr12-23779608261476493] Cheon J. Kim H. Kim D. H. (2024). Healthcare providers’ communication experience in the pediatric intensive care unit: a phenomenological study. BMC Health Services Research, 24(1), Article 956. 10.1186/s12913-024-11394-1 PMC11337589 39164683

[bibr13-23779608261476493] Cho I. Y. Hong S. H. Yun J. Y. (2025). Factors affecting pediatric nurses’ development of partnerships with parents of hospitalized children: An evaluation based on the stress-coping adaptation model. Journal of Child Health Care, 29(1), 53–66. 10.1177/13674935231174501 37217195

[bibr14-23779608261476493] Chong Q. Zhan Z. Peng M. Li X. (2025). The Relationship Between Clinical Communication Skills of Pediatric Nurses and Children’s Trust in Nurses: The Mediating Role of Emotional Intelligence. Nursing: Research and Reviews, 15, 79–89. 10.2147/nrr.s522797

[bibr15-23779608261476493] Claridge A. M. Powell O. J. (2023). Children’s experiences of stress and coping during hospitalization: A mixed-methods examination. J Child Health Care, 27(4), 531–546. 10.1177/13674935221078060 35324345

[bibr16-23779608261476493] Costello M. Rusell K. Coventry T. (2021). Examining the average scores of nursing teamwork subscales in an acute private medical ward. BMC Nursing, 20(1), Article 84. 10.1186/s12912-021-00609-z PMC8166033 34059037

[bibr17-23779608261476493] Emeriaud G. Pons-Òdena M. Bhalla A. K. Shein S. L. Killien E. Y. Modesto i Alapont V. Rowan C. Baudin F. Lin J. C. Grégoire G. Napolitano N. Mayordomo-Colunga J. Diaz F. Cruces P. Medina A. Smith L. Khemani R. G. Pediatric Acute Respiratory Distress Syndrome Incidence Epidemiology Investigators Pediatric Acute Lung Injury Sepsis Investigators Network . (2023). Noninvasive Ventilation for Pediatric Acute Respiratory Distress Syndrome: Experience From the 2016/2017 Pediatric Acute Respiratory Distress Syndrome Incidence and Epidemiology Prospective Cohort Study*. Pediatric Critical Care Medicine, 24(9), 715–726. 10.1097/pcc.0000000000003281 PMC10524424 37255352

[bibr18-23779608261476493] Feng Y. Wang J. Yin Y. Florez I. D. Li Y. Estill J. Ozaki A. Wang X. Hara A. Tao Z. Shao W. Wu Z. Ge L. Chen Y. (2025). Non-invasive respiratory support in pediatric patients with acute hypoxemic respiratory failure: a systematic review and network meta-analysis of randomized controlled trials. eClinicalMedicine, 89, 103550. 10.1016/j.eclinm.2025.103550 PMC12547257 41140447

[bibr19-23779608261476493] Kuzucu S. Türktaş İ. Bedir Demirdağ T. Polat M. Tezer H. Tapısız A. Karakaş N. M. Aslan A. T. Tüzün H. (2025). Acute Bronchiolitis To Pediatric Inpatient Clinic In Patients Under 2 Years Old, Installed with Diagnosis Investigation of the Relationship of Bronchiolitis and Asthma. Gazi Medical Journal, 36(1), 16–23. 10.12996/gmj.2024.4060

[bibr20-23779608261476493] MacKay L. J. Chang U. Kreiter E. Nickel E. Kamke J. Bahia R. Shantz S. Meyerhoff H. (2024). Exploration of trust between pediatric nurses and children with a medical diagnosis and their caregivers on inpatient care units: A scoping review. Journal of Pediatric Nursing: Nursing Care of Children and Families, 78, e1–e30. 10.1016/j.pedn.2024.05.030 39085007

[bibr21-23779608261476493] Malterud K. (2025). Kvalitative Forskningsmetoder for Medisin og Helsefag. Universitetsforlaget.

[bibr22-23779608261476493] Masterson K. Connolly M. Alexander D. Brenner M. (2024). Voice of the nurse in paediatric intensive care: a scoping review. BMJ Open, 14(12), Article e082175. 10.1136/bmjopen-2023-082175 PMC11667362 39806654

[bibr23-23779608261476493] Panetti B. Bucci I. Di Ludovico A. Pellegrino G. M. Di Filippo P. Di Pillo S. Chiarelli F. Attanasi M. Sferrazza Papa G. F. (2024). Acute Respiratory Failure in Children: A Clinical Update on Diagnosis. Children (Basel), 11(10), Article 1232. 10.3390/children11101232 PMC11506303 39457197

[bibr24-23779608261476493] Polit D. F. Beck C. T. (2021). Nursing research: Generating and Assessing Evidence for Nursing Practice (11th ed.). Wolters Kluwer.

[bibr25-23779608261476493] Poole E. I. Ryan M. Walls M. Slumkoski C. Curran J. A. Seabrook J. A. Foster J. R. (2024). “I want to be there. I have to be there.”: Parents’ perceived barriers and facilitators to bedside presence in the pediatric intensive care unit [Original Research]. Frontiers in Pediatrics, 11, 1308682. 10.3389/fped.2023.1308682 PMC10800939 38259595

[bibr26-23779608261476493] Sabetsarvestani R. Geçkil E. (2024). A meta-synthesis of the experience of paediatric nurses in communication with children. Journal of Advanced Nursing, 80(9), 3577–3592. 10.1111/jan.16072 38258623

[bibr27-23779608261476493] Sajid M. Mushtaq B. Abdul Jabbar U. Momna R. Anees J. Siddiqi A. Sohaib M. Khan F. F. Bin Aqeel S. T. Zaidi A. (2026). Efficacy of Non-invasive Ventilation Compared to Intubation in Pediatric Acute Respiratory Failure: A Meta-Analysis. Cureus, 18(3), Article e105202. 10.7759/cureus.105202 PMC13075522 41982577

[bibr28-23779608261476493] Tavallali A. G. Jirwe M. Johansson Stark Å. Eckerblad J. (2025). Nurses’ experiences of cross-cultural care encounters in Swedish pediatric hospital care: A qualitative study. Journal of Pediatric Nursing: Nursing Care of Children and Families, 81, 74–82. 10.1016/j.pedn.2025.01.014 39862708

[bibr29-23779608261476493] Tong A. Sainsbury P. Craig J. (2007). Consolidated criteria for reporting qualitative research (COREQ): a 32-item checklist for interviews and focus groups. International Journal for Quality in Health Care, 19(6), 349–357. 10.1093/intqhc/mzm042 17872937

[bibr30-23779608261476493] Zhang H. Wang J. Li C. Liu X. Xiang Q. (2025). Analysis of the correlation between core competencies of pediatric nurses and occupational benefit perception and its influencing factors. Front Pediatr, 13, 1559572. 10.3389/fped.2025.1559572 PMC12146191 40492260

